# General beliefs about medicines among doctors and nurses in out-patient care: a cross-sectional study

**DOI:** 10.1186/1471-2296-10-35

**Published:** 2009-05-18

**Authors:** Ann-Charlotte Mårdby, Ingemar Åkerlind, Tove Hedenrud

**Affiliations:** 1 Department of Public Health and Community Medicine, University of Gothenburg, Göteborg, Sweden; 2 School of Health, Care and Social Welfare, Mälardalen University, Västerås, Sweden

## Abstract

**Background:**

Doctors and nurses are two natural partners in the healthcare team, but they usually differ in their perspectives on how to work for increased health. These professions may also have different beliefs about medicines, a factor important for adherence to medicines. The aim was to explore general beliefs about medicines among doctors and nurses.

**Methods:**

Questionnaires were sent to 306 private practitioners (PPs), 298 general practitioners (GPs) and 303 nurses in the county of Västra Götaland, Sweden. The questionnaire included sociodemographic questions and the general part of the Beliefs about Medicines Questionnaire (BMQ), which measures the beliefs people have about medicines in general. General beliefs about medicines in relation to background variables were explored with independent t-tests and ANOVA analyses. Differences between occupations and influences of interaction variables were analysed with multiple linear regression models for general beliefs about medicines.

**Results:**

The data collection resulted in 616 questionnaires (62.1% PPs; 61.6% GPs; 80.5% nurses). The majority of the PPs and 40% of the GPs were male but most of the nurses were female. The GPs' mean age was 47 years, PPs' 60 years and nurses' 52 years. Few nurses originated from non-Nordic countries while 15% of the PPs and 25% of the GPs did. Nurses saw medicines as more harmful and less beneficial than did PPs and GPs. These differences could not be explained by the included interaction variables. GPs with a Nordic background saw medicines as more beneficial and less harmful than did GPs with a non-Nordic background.

Furthermore, GPs of non-Nordic origin were most likely to believe that medicines were overprescribed by doctors.

**Conclusion:**

Doctors were more positive about medicines than nurses. The differences in beliefs about medicines found between doctors and nurses could not be explained by any of the included interaction variables. These differences in beliefs may be useful in discussions among future and practising doctors and nurses to enhance understanding of each other's profession and teamwork.

## Background

Medical treatment is prescribed to prevent, cure and treat many diseases and is discussed in patient consultations with doctors and nurses. Information provided during these consultations can be influenced by the beliefs among both patients and healthcare providers [1,2]. Beliefs about medicines have been shown to be important for adherence to medicines among patients [3-5]. A low adherence to treatments, which is a problem in chronic illnesses, has been seen to cause both increased hospitalisations [6] and mortality [7].

Compared with other countries, primary care has been less prominent within healthcare in Sweden [8]. For the last few years community health centres, with several healthcare professionals, have become the base for healthcare in Sweden [8]. Doctors and nurses are two natural partners in the healthcare team. Nurses and general practitioners (GPs) within primary care and private practitioners (PPs) meet and treat the same groups of chronically-ill patients. GPs work primarily in teams in primary care while PPs more often work alone in their own clinics. Within Swedish primary care the nurses may include several specialties: nurses, district nurses and midwives. Although Swedish general practitioners do most of the prescribing, some nurses also prescribe medicines within certain therapy areas like dermatology, anaesthesia, nutrition and birth control. Nurses and district nurses at community health centres may also have special areas of expertise, meeting patients and discussing treatment plans: e.g. asthma and diabetes control.

Although both doctors and nurses work to improve their patients' health their perspectives on how to do that still differ [9,10]. Nurses have been seen as caring for patients' emotional and physical needs [11,12], having a more social perspective [13], including the whole person [9], and may be seen as advocates who speak for the patient's needs within the multidisciplinary team [14]. Doctors, on the other hand, may be seen as more clinical, emotionally neutral [13], setting the priorities and making the decisions [11]. The decisions of doctors are traditionally based more on scientific research [9]. Furthermore, doctors are usually seen as the team leaders [11,15]. These professional factors and the fact that the power imbalance occurring in the doctor-patient relationship is less present between the nurse and the patient, may mean that patients find nurses more approachable when communicating about their own health related problem with a healthcare professional.

The different perspectives on patient care could result in differences in beliefs about medicines among doctors and nurses, as well as different ideas about what kind of treatment is suitable for a patient: medical and/or other treatments. In earlier studies, beliefs about medicines have been examined in university students [16,17], pharmacy employees [18,19] and patients [3] in relation to a number of factors. Some may, however, be of greater importance than others. For example age has not been seen to affect beliefs about medicines [3,18]. Differences in beliefs between males and females are still somewhat unclear [3,16,17]. There is an indication that professional experience can influence beliefs [18]. Cultural origin, however, seems to be important [3,16]. Two studies have found that people of European [16] or Nordic origin [3] had more positive views of medicines compared with those of Asian [16] or non-Nordic [3] origin. Results from a Swedish questionnaire study of Swedish medical and nursing students also showed that healthcare education was an important variable for beliefs about medicines [17]. The medical students had more positive beliefs than nursing students had [17]. If there are differences in beliefs about medicines between practising doctors and nurses, the consequences could be the sending of different messages about medicines to the patient or effects on the working relationship if the differences are not acknowledged. No studies have been found, however, which examine the differences in beliefs about medicines between practising doctors and nurses. It is of considerable importance to explore their beliefs about medicines since they prescribe and/or discuss medicines with patients every day.

The aim of this study was to explore general beliefs about medicines among doctors and nurses.

## Methods

### Participants

The study took place in the county of Västra Götaland, Sweden, in 2007. Healthcare professionals who prescribed and/or discussed treatment plans outside hospital care were the target groups in this study: general practitioners and nurses within primary care, and private practitioners. The nursing group consisted of district nurses, midwives and nurses. The GPs and the nurses were randomly selected from the primary care employee lists supplied by the Pharmaceutical Unit in the Department of Health in Västra Götaland: 298 (38%) of available GPs and 303 (16%) of available nurses. In this study we wanted to concentrate on those PPs with a service contract of care with the county of Västra Götaland. In Västra Götaland there were not, however, enough PPs with such a contract to enable a random selection. Instead, all PPs with a service contract were selected. A few of those without a contract with the county also received a questionnaire to ensure that we received enough questionnaires for a viable study. A total of 306 questionnaires were sent out to PPs.

The county of Västra Götaland is a diverse area including Sweden's second largest city, Gothenburg, smaller towns and rural regions. In Västra Götaland 1844 nurses, 778 GPs and 655 PPs were registered as practising professionals when the study was conducted. Of these, 1.4% of the nurses, 61.4% of the GPs and 68.4% of the PPs were male. The nurses in the county had a mean age of 51 years, the GPs 53 years and the PPs 55 years.

### Data collection

The questionnaire included the general part of the Beliefs about Medicines Questionnaire (BMQ) [5,20] and sociodemographic questions: sex, age, country of birth, parents' country of birth, professional occupation and years of professional experience. The attached letter contained written information about the aim of the study, why the questionnaire was sent to the recipient, the researchers' contact information, and confirmed that the questionnaires were voluntary and anonymous. Doctors and nurses who chose to participate in the study returned the questionnaires in the stamped, addressed envelope provided. A reminder was sent to those who had not responded after one month. The identification list including serial number, names and addresses was destroyed after the reminder was sent.

The Beliefs about Medicines Questionnaire (BMQ) was developed in the UK [5,20]. The questionnaire has been translated into Swedish and the English back-translation has been accepted by the original author of the questionnaire. The original BMQ has been validated with good results and tested for its psychometric capacities [5]. The Swedish version of the BMQ has been tested in a pilot study with good face validity and has been used in several studies of different groups [3,17,18]. BMQ has a general and a specific part. In this study the general part of BMQ was used. General BMQ measures the beliefs people have about medicines in general and consists of twelve statements. These statements can be divided into three separate subparts: General-Harm, General-Overuse and General-Benefit. General-Harm measures beliefs about medicines as something harmful. General-Overuse measures peoples' concerns about doctors overprescribing medicines. General-Benefit measures beliefs about the benefits of medicines. BMQ has response categories on a five-point Likert scale: 1 = strongly disagree, 2 = disagree, 3 = uncertain, 4 = agree and 5 = strongly agree.

### Statistical analysis

All statistical analyses were performed with SPSS version 15.0 for Windows. All statements in a specific BMQ subpart were summarised and divided by the number of statements included in the specific subpart, which resulted in a mean ranging from one to five. A higher mean meant a stronger belief in the concept described by that subpart. A BMQ subpart received a missing value if a participant had one or more missing values for any of the included statements. The internal consistency between the statements was tested in the different subparts with Cronbach's alpha analysis. The internal consistency was 0.64 for General-Harm, 0.76 for General-Overuse and 0.69 for General-Benefit. None of the subparts received a higher Cronbach's alpha value if any of the statements were deleted.

Beliefs about medicines owing to background variables were explored among PPs, GPs and nurses. Dichotomous variables were analysed with independent t-tests: males versus females, birth in and birth outside the Nordic countries, and birth of at least one versus no parents outside the Nordic countries. ANOVA (Analysis of Variance) was used to analyse differences in beliefs owing to age and professional experience. Any differences found in any ANOVA tests were analysed with a post hoc test (Tukey's B).

Multiple linear regression models were constructed to analyse any differences between nurses and doctors. Furthermore, interactions between occupation and other background variables for the dependent variables General-Harm, General-Overuse and General-Benefit were also tested. First separate linear regression models were constructed with each variable for all BMQ subparts respectively to see which variables had any influence on the BMQ subparts. Variables of sufficient potential interest to warrant inclusion in the multiple linear regression models had a p-value < 0.20 [21]. In the regression analysis, age and years of professional experience were treated as continuous variables [22]. Second, statistical analyses with relevant p-values were considered when possible interaction variables for occupation were examined. The distribution of the background variables for GPs, PPs and nurses was also considered. In the final step multiple regression models were constructed for each BMQ subpart with all variables of potential interest. The nurses were treated as a reference group in the multiple linear regression models.

This study was carried out in compliance with the Helsinki Declaration and it was approved by the Ethical Committee of the Sahlgrenska Academy, University of Gothenburg, Sweden.

## Results

### Demographics

A total of 616 questionnaires were returned in this cross-sectional study. The response rates in the doctor groups (GPs: n = 182, 61.1%; PPs: n = 190, 62.1%) were lower than in the nurse group (n = 244; 80.5%). Table 1 shows that about 40% of the GPs and 75% of the PPs were male, while most nurses were female. The average age of the GPs was 47 years, the PPs 60 years and the nurses 52 years. GPs and PPs had a wider age range than the nurses. Over 40% of the GPs had worked as doctors for fifteen years or less. It was more common for PPs (50%) and nurses (30%) to have long professional experience (>30 years). Few of the nurses were born outside or had at least one parent born outside the Nordic countries. For the PPs this percentage was up to 15% and for the GPs 25%.

**Table 1:** Background variables for included doctors and nurses

	**Private practitioners** n (%)	**General practitioners** n (%)	**Nurses** n (%)
Total	190	182	244

**Sex**	n = 189	n = 182	n = 244

Male	142 (75.1)	74 (40.7)	5 (2.0)
Female	47 (24.9)	108 (59.3)	239 (98.0)

**Age in years**			

Mean	60	47	52
SD	7.8	11.0	7.0
Range	39–87	28–75	39–66

Age in years*	n = 187	n = 182	n = 243

≤ 40	2 (1.1)	54 (29.7)	11 (4.5)
41–50	10 (5.3)	48 (26.4)	89 (36.6)
51–60	84 (44.9)	57 (31.3)	103 (42.4)
≥ 61	91 (48.7)	23 (12.6)	40 (16.5)

**Birth area**	n = 187	n = 182	n = 243

Nordic countries	162 (86.6)	139 (76.4)	243 (100.0)
World	17 (13.4)	43 (23.6)	0

**Parents born outside Nordic countries**	n = 185	n = 181	n = 243

0	155 (83.8)	132 (72.9)	233 (95.9)
1	7 (3.8)	5 (2.8)	9 (3.7)
2	23 (12.4)	44 (24.3)	1 (0.4)

Professional experience in years *	n = 182	n = 174	n = 241

≤ 15	6 (3.3)	75 (43.1)	36 (14.9)
16–30	85 (46.7)	72 (41.4)	134 (55.6)
≥ 31	91 (50.0)	27 (15.5)	71 (29.5)

* Displayed in table as categorised variables, but in questionnaire as continuous variables.

The nurses who participated in the study represented the nurses in Västra Götaland well in terms of age and sex. The participating GPs were younger than the GPs in Västra Götaland considered as a whole and fewer males participated. The participating PPs were older and more males participated compared with all PPs in the county of Västra Götaland.

### General beliefs about medicines among doctors and nurses

The data were normally distributed. No significant differences in general beliefs about medicines were found owing to age for any professional category or any other background variable for the nurses. The independent t-tests showed that male PPs saw medicines as more harmful than did female PPs (Table 2). Among all the doctors, GPs as well as PPs, those who stated non-Nordic heritage believed medicines to be more harmful than did doctors of Nordic heritage. Moreover, GPs stating Nordic heritage also saw medicines as more beneficial and less likely to be overprescribed by doctors compared with GPs of non-Nordic heritage. Furthermore, the ANOVA showed that GPs with few years of professional experience were more likely to believe medicines to be overprescribed by doctors than did GPs who had considerable professional experience.

**Table 2:** Doctors' and nurses' general beliefs about medicines in terms of background variables

	**General-Harm** (SD)	**General-Benefit** (SD)	**General-Overuse** (SD)
**Sex **#			

Private practitioners			
Female	1.53 (0.45) *	4.45 (0.44)	2.93 (0.93)
Male	1.75 (0.53)	4.36 (0.53)	2.75 (0.63)
General practitioners			
Female	1.73 (0.58)	4.34 (0.49)	3.07 (0.78)
Male	1.81 (0.53)	4.20 (0.47)	3.16 (0.78)
Nurses			
Female	1.91 (0.53)	4.19 (0.50)	3.49 (0.77)
Male	2.24 (1.02)	3.90 (1.18)	3.73 (1.14)

**Own birth area **#			

Private practitioners			
Nordic countries	1.65 (0.48) *	4.41 (0.49)	2.87 (0.86)
World	1.91 (0.65)	4.26 (0.57)	2.99 (0.92)
General practitioners			
Nordic countries	1.67 (0.49) ***	4.39 (0.42) ***	3.00 (0.75) **
World	2.05 (0.65)	3.93 (0.52)	3.46 (0.74)

**Parents birth area **#			

Private practitioners			
2 born within Nordic countries	1.65 (0.49) *	4.40 (0.50)	2.85 (0.86)
≥1 born outside Nordic countries	1.88 (0.61)	4.28 (0.54)	3.02 (0.90)
General practitioners			
2 born within Nordic countries	1.67 (0.50) **	4.40 (0.42) *	3.01 (0.75) ***
≥1 born outside Nordic countries	1.97 (0.55)	3.95 (0.49)	3.36 (0.75)
Nurses			
2 born within Nordic countries	1.92 (0.53)	4.17 (0.51)	3.48 (0.76)
≥1 born outside Nordic countries	1.92 (0.78)	4.38 (0.73)	3.73 (1.06)

**Professional experience in years **¤^ψ^			

Private practitioners			
≤ 15	1.33 (0.21)	4.50 (0.39)	3.33 (0.99)
16–30	1.71 (0.56)	4.38 (0.49)	2.95 (0.87)
≥ 31	1.69 (0.48)	4.41 (0.52)	2.80 (0.85)
General practitioners			
≤ 15	1.76 (0.60)	4.26 (0.52)	3.23 (0.70) ^a^, *
16–30	1.69 (0.47)	4.29 (0.40)	3.02 (0.74)
≥ 31	1.82 (0.64)	4.31 (0.59)	2.83 (0.87) ^a^
Nurses			
≤ 15	1.81 (0.43)	4.28 (0.40)	3.30 (0.85)
16–30	1.96 (0.58)	4.17 (0.54)	3.55 (0.80)
≥ 31	1.87 (0.53)	4.17 (0.52)	3.49 (0.66)

ψ treated as categorical variables; Missing values not included; Variables without representatives in a category or showed no significance were not included in Table 2;

*** p < 0.001, ** p < 0.01, * p < 0.05; # independent t-test; ¤ ANOVA was made to test any differences: pairs within a subpart and year marked with a are significantly different from each other (post hoc test: Tukey's B)

### Differences in beliefs about medicines between doctors and nurses

According to separate linear regression models, variables of potential interest for further analysis were occupation, and own and parents' birth area for General-Harm and General-Benefit. For General-Overuse these variables were occupation, sex, age and professional experience. Results from statistical analysis and the distribution of the variables showed that birth area*occupation, and parents birth area*occupation were potential interaction variables in the models for General-Harm and General-Benefit. For the General-Overuse model these were: sex*occupation, age*occupation and professional experience*occupation. No nurse was born outside the Nordic countries, so this variable was excluded from further analysis.

The multiple linear regression models for the BMQ subparts can be viewed in Table 3, with nurses as reference group. The GPs and PPs did believe medicines to be more beneficial and less harmful than did the nurses. None of the included background variables showed any interactions with occupation for General-Harm. For General-Benefit one interaction variable was significant: GPs with at least one parent born outside the Nordic countries did see medicines as less beneficial than nurses with at least one parent born outside the Nordic countries. The difference in beneficial beliefs between the GPs and the nurses, however, remained. For General-Overuse, no significant differences were found between the nurses and the doctors, indicating some interaction between occupation and the other included variables.

**Table 3:** Three linear regression models including a BMQ subpart (dependent variables) and background variables (independent variables)

**General-Harm**	**B (SD)**	**p-value**
General practitioners	-0.240 (0.059)	<0.001
Private practitioners	-0.267 (0.057)	<0.001
At least 1 parent born outside the Nordic countries	0.003 (0.171)	0.985
At least 1 parent born outside the Nordic countries*GP	0.252 (0.193)	0.192
At least 1 parent born outside the Nordic countries*PP	0.227 (0.201)	0.259

**General-Benefit**	**B (SD)**	**p-value**

General practitioners	0.217 (0.054)	<0.001
Private practitioners	0.232 (0.052)	<0.001
At least 1 parent born outside the Nordic countries	0.204 (0.159)	0.202
At least 1 parent born outside the Nordic countries*GP	-0.577 (0.180)	0.001
At least 1 parent born outside the Nordic countries*PP	-0.331 (0.188)	0.079

**General-Overuse**	**B (SD)**	**p-value**

General practitioners	0.337 (0.805)	0.676
Private practitioners	-0.476 (0.814)	0.559
Female	-0.285 (0.404)	0.481
Female*general practitioners	0.208 (0.423)	0.623
Female*private practitioners	0.085 (0.427)	0.843
Age in years	0.012 (0.011)	0.288
Age in years*GP	-0.018 (0.019)	0.345
Age in years*PP	0.005 (0.016)	0.743
Professional experience in years	-0.001 (0.008)	0.902
Professional experience in years*GP	-0.005 (0.017)	0.747
Professional experience in years*PP	-0.025 (0.015)	0.085

Nurses as the reference group; GP = general practitioners; PP = private practitioners

Missing values not included

Excluded from all three multiple linear regression models: born within/outside Nordic countries

## Discussion

This study showed differences in general beliefs about medicines between the participating doctors and the nurses. GPs and PPs believed there to be more benefits and less harmful effects of medicines compared with nurses. Birth area of the GPs parents was a statistically significant interaction variable for occupation in General-Benefit. The interaction variables included in this study have been shown to be important for general beliefs about medicines in pharmacy clients [3] and university students [16,17]. None of the included background variables, however, could fully explain why these differences in beliefs existed between doctors and nurses. It is possible that other variables not included in this study are of significance for general beliefs about medicines among doctors and nurses. Type of education may be one possible factor contributing to the different perspectives on medicines. A recent Swedish questionnaire study showed that medical students demonstrated more salient development of their positive beliefs about medicines than nursing students did [17]. The observed differences in beliefs about medicines found between doctors and nurses are not surprising, but are, nevertheless, important. Both doctors and nurses are part of the healthcare team that has daily contact with patients to discuss medicines. One aspect of different beliefs about medicines may be different opinions about what treatment a patient should receive and the consequent provision of different information to the patient about the treatment: the patient could then have difficulties evaluating the treatment. Furthermore, it has been perceived by Swedish doctors that if the same message is delivered by different professions making up the healthcare team the patient adheres to the message better [15].

Holding different beliefs may not always be negative. Since differences in beliefs about medicines have been seen both between future [17] and practising doctors and nurses, those differences may be used in a creative way in the teamwork process. Today teamwork in primary care has been extended to include other healthcare professions: e.g. physiotherapists, psychologists and social workers [15,23]. These professions might have different beliefs about medicines compared with doctors and nurses. This is yet to be examined. To be able to come up with creative solutions in patient care it is important to be open about each other's beliefs, see things from other professionals' perspective and not take the beliefs of others for granted [10]. Storch and colleagues suggested regular meetings where ethics and collaboration should be discussed [9]. If these also include discussions about beliefs about medicines this may contribute to increased understanding and improved teamwork. It is also important to incorporate this during the education of future doctors and nurses. Trust and understanding in teamwork are important for patient safety [9,24].

Interactions occurred between occupation, sex, age and years of professional experience in the multiple linear regression model for General-Overuse. These interactions erased the initial differences between doctors and nurses. Further research is needed to examine exactly how these variables interact with occupation for General-Overuse.

When the beliefs about medicines were explored further among the included nurses, GPs and PPs it was apparent that doctors' own or their parents' birth area were of most importance. GPs with a Nordic background were overall more positive to medicines than the GPs who had a non-Nordic background. A similar result could be observed for the PPs, although only General-Harm was significant. The importance of origin for general beliefs about medicines confirms the results seen in studies with other populations including students [16] and pharmacy clients [3]. The results of the present study, however, add a new dimension since a doctor's beliefs about medicines may influence the patient consultation. About 50% of all prescribed medicines are not used as the prescriber intended [6,25]. Large amounts of medicines are therefore returned and then destroyed at the pharmacies [26,27]. Perhaps patients receive more prescriptions for medicines when consulting doctors with a Nordic rather than a non-Nordic background. Additional studies are needed, however, to verify any association between beliefs about medicines and prescribing patterns.

It is possible that there are differences in general beliefs about medicines between patients, doctors and nurses. This is the conclusion drawn by a Swedish thesis [28]. Patients were more likely than both doctors and nurses to believe that medicines are more harmful [28]. A Finnish questionnaire study observed that over 40% of the included pharmacists did not agree that their own beliefs could be a barrier in patient counselling [29]. If this is the case for nurses and doctors as well it could have an impact on healthcare. Research shows that doctors and nurses still dominate the communication situation [30-34]. Furthermore, patients are not always encouraged to discuss medicines [34]. It is important for doctors and nurses to stimulate communication about the patient's beliefs about medicines and be aware that patients' beliefs about medicines may differ from those of both doctors and nurses. This could increase the likelihood of patient-centred care, a biopsychosocial way of patient care [35] shown to be associated with health [35-37] and increased adherence to medicines [35,38].

In this study, Cronbach's alpha was used to measure internal consistency. A low Cronbach's alpha value means that the statements do not belong in the same subpart [39]. There is not, however, any consistency in the literature about a lower acceptable limit: 0.70 [39], 0.60 [40] and as low as 0.50 [39]. The conclusions of this study ought to be drawn in the light of the above.

Some of the doctors and nurses seemed to have difficulties generalising the statements on the BMQ. These participants possibly saw certain statements or the whole questionnaire as a test of knowledge. This resulted in some participants giving two opposite answers on a few statements. These statements were treated as missing values.

The questionnaires and information were sent out by mail to doctors and nurses practising medicine and care in the county of Västra Götaland, Sweden. This could have been perceived as an invasion of privacy by the receivers. The questionnaire, however, included detailed information showing that the Pharmaceutical Unit in the Department of Health in the county of Västra Götaland was responsible for handling the addresses of the doctors and the nurses while the researchers and the University of Gothenburg were responsible for the actual study.

This is a cross-sectional study and the possibility of drawing any conclusions regarding causality is, of course, limited.

It is likely that the results from this study can, at least, be partly generalised to a Swedish level. The participating nurses did not differ from the population of nurses in the county of Västra Götaland according to age or sex. The PPs who participated did not differ much in sex but had a higher mean age than the total population of PPs in the county of Västra Götaland. There were fewer male and slightly younger GPs in this study compared with all GPs in the county. According to previous studies, age is not a significant factor for beliefs [3,18]. Present results and previous studies [3,16] indicate that sex probably has only slight effect on beliefs. Furthermore, the study included doctors and nurses from a large county in Sweden which contains the second largest city, smaller cities and rural parts. It was not, however, possible to obtain information on the percentage of nurses and doctors who were of non-Nordic origin on a Swedish level. Stated origin was relevant to general beliefs about medicines for doctors. If the percentage of doctors with a non-Nordic origin differs considerably on a Swedish level from the percentage in this study, the generalisability of these results may be questionable.

## Conclusion

Nurses saw medicines as more harmful and less beneficial than did general practitioners and private practitioners. None of the included interaction variables could explain the differences in harmful and beneficial beliefs found between doctors and nurses. These differences in beliefs may be brought out in discussion groups comprising members of both professions to create a deeper understanding of one another's professions and improved teamwork. Inclusion of these discussions in the education of future doctors and nurses is another possibility. More studies are needed to explore the beliefs about medicines of other professionals in the healthcare team. Doctors with a Nordic background were more positive about medicines than doctors with a non-Nordic background. Any association between beliefs about medicines and prescribing patterns needs to be investigated in future studies.

## Competing interests

The authors declare that they have no competing interests.

## Authors' contributions

A-CM planned, collected, analysed and interpreted the data of this study and wrote the manuscript. IÅ and TH were involved in planning the study, in interpretation of the data and in writing this manuscript. All authors read and approved the final manuscript.

## Pre-publication history

The pre-publication history for this paper can be accessed here:


